# Novel Mechanism of Arenavirus-Induced Liver Pathology

**DOI:** 10.1371/journal.pone.0122839

**Published:** 2015-03-30

**Authors:** Juliane I. Beier, Jenny D. Jokinen, Gretchen E. Holz, Patrick S. Whang, Amah M. Martin, Nikole L. Warner, Gavin E. Arteel, Igor S. Lukashevich

**Affiliations:** 1 Department of Pharmacology and Toxicology, University of Louisville Health Sciences Center, Louisville, Kentucky, United States of America; 2 Department of Microbiology and Immunology, University of Louisville Health Sciences Center, Louisville, Kentucky, United States of America; 3 Center for Predictive Medicine for Biodefense and Emerging Infectious Diseases, Louisville, Kentucky, United States of America; The Scripps Research Institute, UNITED STATES

## Abstract

Viral hemorrhagic fevers (VHFs) encompass a group of diseases with cardinal symptoms of fever, hemorrhage, and shock. The liver is a critical mediator of VHF disease pathogenesis and high levels of ALT/AST transaminases in plasma correlate with poor prognosis. In fact, Lassa Fever (LF), the most prevalent VHF in Africa, was initially clinically described as hepatitis. Previous studies in non-human primate (NHP) models also correlated LF pathogenesis with a robust proliferative response in the liver. The purpose of the current study was to gain insight into the mechanism of liver injury and to determine the potential role of proliferation in LF pathogenesis. C57Bl/6J mice were infected with either the pathogenic (for NHPs) strain of lymphocytic choriomeningitis virus (LCMV, the prototypic arenavirus), LCMV-WE, or with the non-pathogenic strain, LCMV-ARM. As expected, LCMV-WE, but not ARM, caused a hepatitis-like infection. LCMV-WE also induced a robust increase in the number of actively cycling hepatocytes. Despite this increase in proliferation, there was no significant difference in liver size between LCMV-WE and LCMV-ARM, suggesting that cell cycle was incomplete. Indeed, cells appeared arrested in the G_1_ phase and LCMV-WE infection increased the number of hepatocytes that were simultaneously stained for proliferation and apoptosis. LCMV-WE infection also induced expression of a non-conventional virus receptor, AXL-1, from the TAM (TYRO3/AXL/MERTK) family of receptor tyrosine kinases and this expression correlated with proliferation. Taken together, these results shed new light on the mechanism of liver involvement in VHF pathogenesis. Specifically, it is hypothesized that the induction of hepatocyte proliferation contributes to expansion of the infection to parenchymal cells. Elevated levels of plasma transaminases are likely explained, at least in part, by abortive cell cycle arrest induced by the infection. These results may lead to the development of new therapies to prevent VHF progression.

## Introduction

Viral infections targeting the liver remain a major cause of human morbidity and mortality and can induce fatty liver, fibrosis, and hepatocellular carcinoma. Viral hepatitis is the leading cause of primary liver cancer and the most common indication for liver transplantation [1]. Virus infections causing acute liver failure can be separated into two groups: (i) primary hepatitis (e.g., hepatitis A, B, and C virus infections); and (ii) hepatitis occurring as part of systemic infections (e.g., viral hemorrhagic fevers, VHFs). Virus-specific CD8+ T cells play key roles in the pathogenesis of viral hepatitis; they are involved in rapid activation to effectively control virus replication and persistence. On the other hand, virus-specific T cells target infected hepatocytes during acute or persistent infection, which causes liver injury. Better understanding of the interplay between viral infection, immune surveillance and liver function is a key to preventing the injury that these infections cause.

Lymphocytic choriomeningitis virus (LCMV), the prototypic arenavirus, is hosted by the house mouse, *Mus musculus*, distributed worldwide and causes chronic asymptomatic infections in natural hosts. Experimental LCMV infection of mice is a widely used model to study basic virology and immunological mechanisms of interaction between viruses and their natural hosts. Many fundamental discoveries were made using this model (e.g., MHC restriction, T cell memory, persistent infections, T cell exhaustion, immunopathology, etc.) [2–4]. LCMV belongs to the Old World (or LCMV-LASV) sub-group of the *Arenaviridae*. Arenaviruses are enveloped viruses with a bi-segmented negative strand RNA genome [5]. Each genomic RNA segment, L and S, encodes two gene products. The L RNA encodes the viral polymerase (RdRp) and matrix protein (Z), whereas the S RNA encodes the viral surface glycoproteins (GP) and nucleoprotein (NP). In New World primates of the family *Callitrichidae* (marmosets, tamarinds, and Goeldi's monkeys), LCMV causes highly fatal disease, Callitrichid hepatitis (CH), with elevated liver enzymes, jaundice, and sometimes hemorrhage. In experimentally infected rhesus macaques, LCMV-WE causes fatal liver disease [6–9] resembling Lassa Fever (LF) which was initially described as Lassa virus (LASV) hepatitis [10,11].

LASV is the most prominent human pathogen of the *Arenaviridae* with the highest human impact of any of the hemorrhagic fever viruses (with the exception of Dengue virus) [12–16]. In addition to arenaviruses, causative agents of VHFs belong to *Bunyaviridae* (e.g., Crimean Congo HFV virus and Rift valley fever virus, RVFV), *Filoviridae* (Ebola virus and Marburg virus), *Flaviviridae* (e.g., Dengue virus, DENV and Yellow fever virus, YFV), and *Rhabdoviridae* (Bas-Congo virus, BASV) [17]. Although these infections are caused by taxonomically diverse viruses, they share some common mechanisms of infection and pathogenicity in humans [14,18–23]. For many VHFs, the liver is one of the most affected organs participating in systemic breakdown resulting in vascular abnormalities, bleeding, multi-organ failure, and endotoxin-like shock [10,23–25].

Analysis of available LCMV strains showed that these viruses are highly diverse, genetically and biologically [26,27]. Armstrong (ARM) and WE are commonly used strains of LCMV in animal models. LCMV-ARM (strain 53b) is highly adapted to tissue culture and murine models, and is widely considered to be neurotropic. LCMV-WE (strain 54) replicates primarily in macrophages, epithelial and parenchymal cells and is generally described as viscerotropic. Both strains share high homology at the nucleotide (94%) and amino acid (84%) levels and carry the same H-2b-restricted CTL epitopes. Nevertheless, infection with WE, but not ARM, induces liver disease in mice and fatal LF-like hepatitis in rhesus macaques [6–9,28,29]. Previously we showed that in rhesus macaques infected with LCMV-WE, but not with LCMV-ARM, 25–40% of hepatocyte nuclei were positively stained for proliferation antigen Ki-67. Notably, the elevated levels of pro-inflammatory cytokines and their receptors in infected monkeys resembled a priming stage of hepatocyte hyperplasia after surgical or toxic injury [7].

With some limitations, murine infection with LCMV has been used as a model to mimic viral liver infections in humans [28,30]. Similar to HBV and HCV, LCMV is not cytopathic. There is a solid body of evidence that cytolytic CD8+ T cells (CTL)-induced liver pathology caused by hepatotropic viruses and LCMV correlates with the number of virus-infected hepatocytes [3,28,31,32]. However, recent studies showed that liver pathology in this model could not be completely attributed to CTL responses (see Discussion; [39–44]). For example, although infection with LCMV-WE caused liver damage and LCMV-ARM did not, there were no differences in LCMV-specific CD8+ T cells between WE- and ARM-infected mice [33]. Furthermore, based on a functional pathway analysis of differentially expressed genes [34], virulent LCMV-WE had a broader effect on liver cell function than did infection with non-virulent LCMV-ARM. The purpose of the current study was to build on recent observations in non-human primates by examining LCMV infection in a murine model and specifically to address the potential role of virus-induced proliferation in liver injury and pathogenesis.

## Materials and Methods

### Cells and Viruses

Vero E6 cells (ATCC CRL-1586) were maintained in 1× DMEM, supplemented with 10% FBS and with 1% antibiotic-antimycotic in a humidified chamber at 37°C, 5% CO_2_. Cells were infected with LCMV-ARM (strain 53b) or LCMV-WE (strain 54) at a multiplicity of infection (MOI) of 1 PFU/cell for 1 hour at 37°C [35]. Virus stocks (5 x 10^6^–1 x10^7^PFU/ml) were harvested in cell-free medium at stored at -80°C until use. The stocks did not contain detectable levels of cytokine as measured by multiplex immunoassay (Bio-Rad). Viral titers were determined by standard plaque assays. Briefly, a monolayer of Vero E6 cells was inoculated with 10^1^-10^6^-fold diluted virus in a 6-well culture plate for 1 hour in a humidified chamber at 37°C, 5% CO_2_ with periodic rocking. An overlay solution containing 1× MEM, 2% FBS, 0.5% agarose was applied to each well and incubated for 5 days. Cells were fixed with 42% paraformeldehyde and the agarose was removed. Cells were stained with 0.1% crystal violet.

### Animals and Treatments

Animals were housed in a pathogen-free ABSL-3 level barrier facility accredited by the Association for Assessment and Accreditation of Laboratory Animal Care and procedures were approved by the University of Louisville Institutional Animal Care and Use Committee. Eight week old female C57BL/6J (H-2^b^, B6) mice were purchased from Jackson Laboratory (Bar Harbor, ME). Food and tap water were provided ad libitum prior to experimentation. Mice were intravenously infected with LCMV-WE or with LCMV-ARM, 1×10^6^ PFU in 0.3 ml of PBS. LCMV infection of mice did not induce significant health concerns. During the 1^st^ week after infection, mice had no sign of disease. Signs of general malaise appeared between day 7 and day 10. Animals moved slowly, were hunched and had ruffled fur in concordance with previous observations [28]. All animals were in relatively good condition on day 12, when the experiment was terminated. On day 0, 4, 8, and 12, four animals per group were sacrificed using chemical euthanasia (inhalation of CO_2_); death of animals was ensured by cervical dislocation. Animals sacrificed on day 0 were not infected and were used as a control group. Blood samples (approximately 0.5 mL) were collected from the vena cava and transferred into a tube containing EDTA and aprotinin (1 mg/mL). The liver was isolated from surrounding tissue and carefully removed from the peritoneum. Approximately 100 mg of the same lobe from each animal was collected into a screw top tube filled to 1/3 with glass beads and with 1 mL of ice cold RNA-Stat60 for further RNA isolation. Another 1–2 pieces of liver (approximately 200 mg) were frozen immediately in liquid nitrogen, while others were fixed in 10% neutral buffered formalin for later histological analyses.

In some experiments, seventy percent partial hepatectomies were performed as described by Greene and Puder [36], with minor modifications. Briefly, anesthesia was initiated and maintained with isoflurane (2% with 2L/min O_2_ flow). A vertical midline incision was made in the skin of the abdomen from the xiphoid cartilage extending to the mid-abdomen. The falciform ligament above the median lobe was cut just proximal to the superior vena cava. The left lateral and median lobes were ligated and cut away from the liver using microsurgery scissors. The mouse was given 5 ml sterile saline subcutaneously on the back after closing the abdomen to account for fluid loss during surgery.

### Histological Studies, Immunofluorescence and Immunohistochemistry

For light microscopy, tissue samples were fixed in buffered formalin, dehydrated, embedded in paraffin, sliced into 5-μm sections, placed on Fisher Plus slides and stained with hematoxylin and eosin (H&E). Hepatocyte hyperplasia was assessed via Ki-67 [7] and proliferating-cell-nuclear-antigen (PCNA) staining [37,38]. Cell cycle progression (per 1,000 hepatocytes) was estimated using PCNA staining patterns and cell morphology as described previously [37,38]. Briefly, G_0_ hepatocytes do not stain for PCNA; G_1_ cells are lightly stained in the nucleus; cells in the S-phase have darkly stained cell nuclei; G_2_ cells have a cytoplasmic staining pattern with or without nuclear staining; M cells have diffuse cytoplasmic and deep blue chromosomal staining and a clear presence of mitotic bodies [37,38].

For immunofluorescent (IFA) detection of LCMV, paraffin embedded liver sections were deparaffinized and blocked with PBS containing 10% goat serum, 0.1% Triton-X-100 and 0.05% Tween 20 for 30 min, followed by incubation overnight at 4°C with an LCMV antibody (Abcam, Cambridge, MA). Sections were washed with PBS and incubated for 2 h with a secondary antibody conjugated to Alexa 488 (Invitrogen, Carlsbad, CA). Sections were incubated with Trypan blue (250 μg/ml in PBS pH 4.4) for 1 min, washed with PBS and mounted with medium containing 1.5 μg/ml DAPI (VECTASHIELD w/DAPI; Vectastain, Torrance, CA). Staining for a marker of oxidative damage and lipid peroxidation (4HNE) was performed using an anti-4-HNE IgG monoclonal antibody (Alpha diagnostics, San Antonio, TX). Oval cells were stained using oval cell marker antibody (OV-6, sc-101863) from Santa Cruz Biotechnology, Inc. Cells undergoing apoptosis were detected in situ by TUNEL using a commercially available kit (Millipore, Billerica, MA). Briefly, tissue sections were digested with proteinase K for 15 min at 37°C and labeled using 10 U of TdT and 17 mM biotin-16-dUTP in 50 ml of TdT buffer. Labeled cells were detected either for bright field by using HRP-conjugated streptavidin (1:100) and bound HRP was visualized by the substrate 3-amino-9-ethylcarbazole or for immunofluorescence by using an anti-digoxigenin antibody with a rhodamine fluorochrome and mounted with mounting medium containing 1.5 μg/ml DAPI (VECTASHIELD w/DAPI; Vectastain, Torrance,CA).

Portal expansion was quantitated as has been described previously [39]. Briefly, the total (A_TOT_) and vessel (A_PV_) area of 10 randomly selected portal regions was determined at 40× using Metamorph image-analysis software (Chester, PA) incorporating a Nikon microscope (Nikon, Melville, NY). For consistency of analysis, portal areas with large (>10×10^–3^ mm^2^), or longitudinally cut, portal venules were avoided. The ratio of cellular to vessel area was calculated using the following equation [(A_TOT_-A_PV_)/A_PV_]. Results were normalized to fold of control animals.

### Detection of Host Proteins in Liver Samples from LCMV-Infected Mice

Liver samples from control and infected animals were homogenized in protein extraction buffer (50mM Tris-HCl pH 7.4, 5mM EDTA, 5 mM EGTA), containing protease and phosphatase inhibitor cocktails (Sigma-Aldrich, St. Louis, MO). Samples were then rocked at 4°C for 4h. Supernatant from each sample was collected and protein concentration was determined using the Bio-Rad DC Protein Assay kit (Bio-Rad Laboratories, Hercules, CA). Extracted protein samples were combined with 4X Laemmli sample buffer (250mM Tris pH 7.4, 10% SDS, 20% 2-mercaptoethanol, 40% glycerol, and 0.01% w/v bromphenol blue) and boiled at 95°C for 5 min. Samples were loaded onto SDS-polyacrylamide gels of 10% and 15% (w/v) acrylamide followed by electrophoresis and Western blotting onto PVDF membranes (Gen Hunter Corporation, Nashville, TN). Primary antibodies against p21 (ab109199, Abcam, Cambridge, MA), AXL (sc-1096, Santa Cruz Biotechnology, Santa Cruz, CA) and GAPDH (sc-25778, Santa Cruz Biotechnology) were used at dilutions 1:1000, 1:750, and 1:2000, respectively. Bands were visualized using horseradish peroxidase-coupled secondary antibodies, an ECL kit (Pierce, Rockford, IL) and Hyperfilm (GE Healthcare, Piscataway, NJ). Densitometric analysis was performed using UN-SCAN-IT gel (Silk Scientific Inc., Orem, UT) software.

### RNA Isolation and Real-Time RT-PCR

RNA extraction and real-time RT-PCR was performed as described previously [40,41]. PCR primers and probes were ordered as commercially available kits (Applied Biosystems, Foster City, CA). Detection of LCMV by qRT/PCR was performed as previously described [6] using virus-specific primers/probes targeting the L RNA of LCMV-ARM 53b (5’CCTTAAAGAGGTGAGAGCATGA, forward; 5’TTTCATTGATATTCTTGGTTAGGTG, reverse; 5’ CAGCCACACCTGGATTCTGTAATTGG, probe) or LCMV-WE (5’CCTGGACTCTGTAATTGGCA, forward; 5’TTACATGCTCAGCAGCACAG, reverse; 5’ TCACAGTGGATTTCACACACAACCAGA, probe). The linearized recombinant plasmids carrying the L RNA segments of both strains were used to make quantitation standards. The standard curves were constructed by plotting copies against the C_T_ values (threshold cycles) at a defined threshold using plasmid standards with high correlation coefficient (R^2^ = 0.988) and high efficiency (>95%). For some host targets, primers/probes were designed using Primer 3 software (Whitehead Institute for Biomedical Research, Cambridge, MA) to cross introns and to ensure that only cDNA, and not genomic DNA, was amplified. The sequence of primers and probes can be provided upon the request. The comparative C_T_ method determines the amount of target, normalized to an endogenous reference (18S) and relative to a calibrator (2^-ΔΔCt^).

### Statistical Analyses

Results are reported as means ± SEM (n = 4–7). ANOVA with Bonferroni’s post-hoc test (for parametric data) or Mann-Whitney Rank Sum test (for nonparametric data) was used for the determination of statistical significance among treatment groups, as appropriate. A *p* value less than 0.05 was selected before the study as the level of significance. Legends to figures included additional details of statistical analysis.

## Results

### LCMV-WE infection in C57BL/6J mice was associated with mild inflammation in liver, oxidative stress and up-regulation of non-conventional LCMV receptors for virus entry

**Fig 1:**
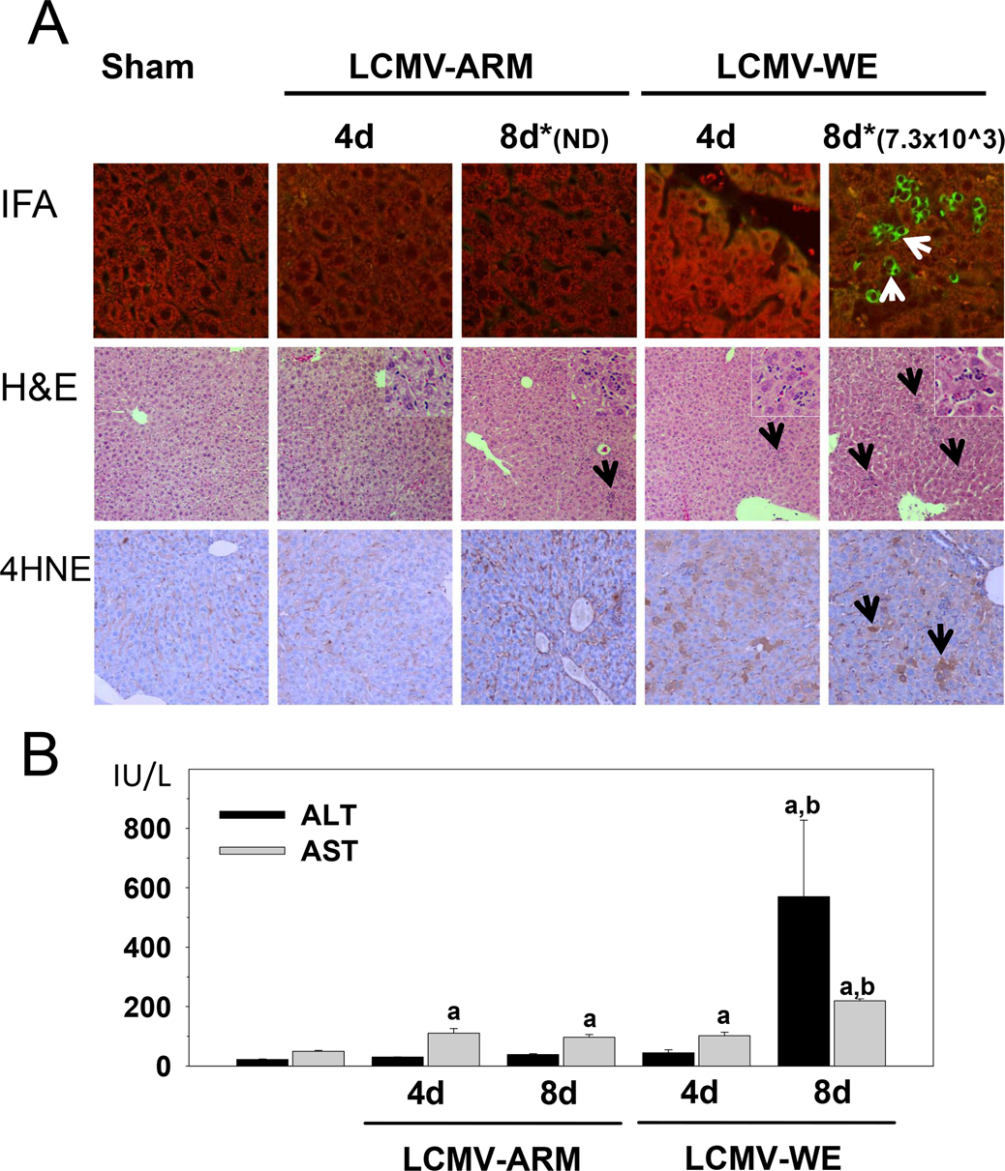
Transient hepatitis in LCMV-WE- infected C57BL/6J mice. Female C57BL/6J mice were divided into two groups and intravenously infected with either LCMV-WE or with LCMV-ARM, 1×10^6^ PFU in 0.3 ml of PBS. Panel A. IFA, staining for LCMV antigen (400x magnification); H&E, staining for hematoxylin and eosin (200x); 4HNE, IHC with a monoclonal anti-4HNE IgG antibody as a marker of oxidative damage and lipid peroxidation (200x). Arrows point to positive staining (IFA and 4HNE) or inflammatory foci (H&E). Insets (1000x magnification) depict inflammatory cell recruitment in LCMV-infected livers. *, numbers above IFA staining panels are viral titer values for LCMV-WE infected liver extracts in PFU/g; ND, not determined, lower than detection limit (<1.2 log_10_ PFU/g, see details in Results). Panel B. Plasma alanine aminotransferase (ALT) and aspartate aminotransferase (AST) were determined as described in Materials and Methods. Data are means ± SEM (n = 4–7).^a^, *P*<0.05 compared with sham infection;^b^, *P*<0.05 compared with LCMV-ARM infection.

We previously reported that the liver was one of the most affected organs in LCMV-infected rhesus macaques. In that study, infection with LCMV-WE, but not LCMV-ARM, negatively impacted biochemical, excretory, and synthetic functions of the liver, concomitant with a rapidly developed fatal LF-like disease [6–9]. As was shown previously, in young adult immunocompetent mice LCMV-WE induced only a mild infection with signs of general malaise even after i.v. inoculation at high dose. These results recapitulate the finding that, in contrast to non-human primates, the disease is rarely fatal in mice [28]. However, similar to infection in rhesus macaques, LCMV-WE induced hepatitis in C57Bl/6J mice. As seen in Fig 1A, LCMV antigen was detected at the peak of the disease, on day 8 after infection, when liver injury was clearly confirmed by elevated serum ALT and AST levels (Fig 1B). In accordance with the transient nature of the LCMV-induced hepatitis in mice, serum aminotransferase levels returned to normal ranges at day 12 after infection (not shown).

In LCMV-WE-infected mice, viral antigen was localized predominantly in hepatocytes and resident macrophages, Kupffer cells (KCs), but was also seen in endothelial cells of the sinusoids (Fig 1A). Light microscopy of H&E-stained sections found disseminated spotty necrosis and foci of mild inflammation seen as mononuclear infiltrates localized predominantly in the periportal zone. These histological signs of hepatitis were found in sections of LCMV-WE-infected mice at day 8 and disappeared by the end of the study (day 12, not shown).

Recent studies showed that oxidative stress impaired the immune response and delayed control of LCMV-WE in mice [42]. This is consistent with our findings in liver sections stained for protein adducts of 4-hydroxynonenal (4HNE), a product of lipid peroxidation (Fig 1A). Whereas the amount of 4HNE adducts was increased with both infections, the magnitude was much stronger after infection with LCMV-WE, especially during earlier stages of infection (day 4).

Mice infected with the same dose of LCMV-ARM did not express any clinical signs of the disease. Serum levels of aminotransferases were only slightly higher than the normal range. Consistent with our previous observations in rhesus macaques [6], LCMV-ARM infection was well controlled in liver tissues. Sensitive qRT/PCR with strain-specific primers showed that viral RNA copies in liver tissues dramatically decreased in LCMV-ARM-infected mice from 5.8±0.63 log_10_ copies of the L genomic segment per gram of tissues (lg RNA copies/g) at day 4, to 3.4±0.51lg RNA copies/g at day 8. This pattern is in accordance with previously published results in this model [33]. In contrast, in LCMV-WE-infected mice viral RNA burden was practically unchanged, 5.66±0.55 and 5.51±0.61 lg RNA copies/g at day 4 and 8, respectively. Although we were able to detect viral RNA in LCMV-ARM-infected liver tissues with strain-specific primers on day 8, plaque assays did not reveal infectious virus particles. In contrast, replication-competent virus was easily detected in LCMV-WE-infected mice (Fig 1A). The difference between viral RNA copies and plaque assay results was likely due to generation of defective-interfering (DI) particles during replication of arenaviruses [43]. Therefore, viral RNA copies do not accurately reflect viral burden (infectious particles) in tissues of infected animals.

Detection of LCMV-WE in hepatocytes (Fig 1A) of experimentally infected mice [28,33] and in rhesus macaques [7,9] is well supported by previous findings in LASV-infected non-human primates [44–48] and in LF patients [11,49]. However, this fact is in conflict with well-documented evidence that mature hepatocytes do not express functionally active (i.e., properly glycosylated alpha-dystroglycan, α-DG), the canonical receptor for LCMV and LASV [50]. Expression of functional α-DG binding to mAb IIH6 in Western blot was detected only in embryonic and early postnatal liver, and was undetectable in hepatocytes of adult animals [51]. These findings suggest a developmental loss of functional α-DG on the surface of hepatocytes due to down-regulation of LARGE and possibly other glycosyltransferases involved in biosynthesis of α-DG [52].

**Fig 2:**
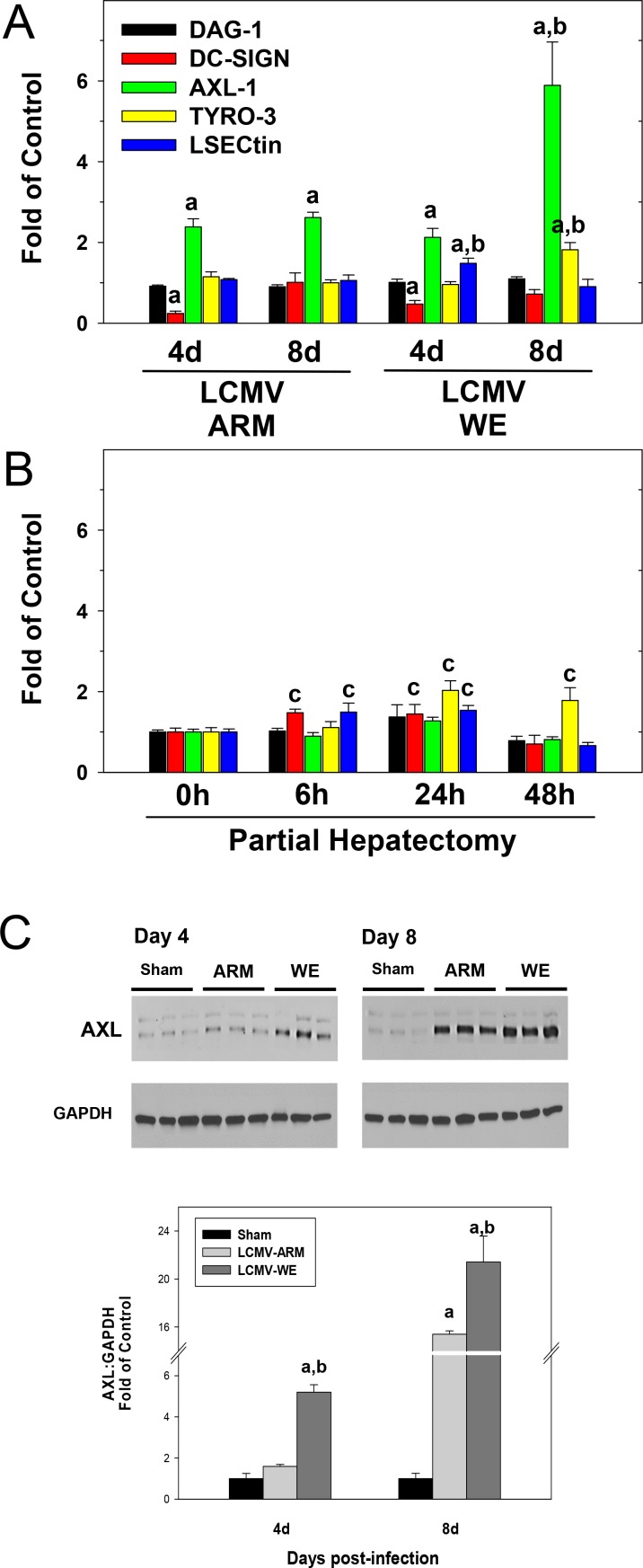
LCMV-WE infection upregulates expression of genes encoding non-conventional receptors for virus entry. Hepatic mRNA was extracted from LCMV-infected mice (Panel A) or from mice after partial hepatectomy (Panel B) and real-time RT-PCR was performed as described in Materials and Methods. Western blot analysis of AXL protein expression (Panel C) was determined as described in Materials and Methods. GAPDH was used as a loading control. Representative bands (upper) and quantitative densitometry (lower) are shown. Quantitative data are means ± SEM (n = 4–7).^a^, *P*<0.05 compared with sham infection;^b^, *P*<0.05 compared with LCMV-ARM infection;^c^, *P*<0.05 compared sham surgery (t = 0; Panel B). Data are representative of two experiments performed with liver extracts collected from three mice in each group.

To test the possibility that recently described “non-conventional” receptors [53–55] for LCMV and LASV were involved in hepatocyte entry, we measured mRNA expression of Axl-1 and Tyro3 (from the TAM family); DC-specific intercellular adhesion molecule 3-grabbing non-integrin, DC-SIGN; and liver and lymph node sinusoidal endothelial calcium-dependent lectin, LSECtin (from the C-type lectin family) in liver from LCMV-infected mice (Fig 2A). Whereas LCMV infection had no effect on *Dag-1* (gene for dystroglycan) or *Dc-sign* mRNA expression, *Tyro-3* and *LSECtin* were induced in livers from LCMV-WE infected mice. LCMV infection by both strains increased the expression of *Axl-1* at 4 and 8 days after infection; the expression of this receptor in livers at day 8 after LCMV-WE infection was ~3-fold higher than in corresponding LCMV-ARM-infected livers. Accordingly, LCMV infection strongly up-regulated Axl protein levels (Fig 2C). Notably, this up-regulation was more robust for LCMV-WE at 4 and 8 days after infection and significantly higher than that for LCMV-ARM infection.

### LCMV-WE infection induced a robust proliferative response in mouse liver

**Fig 3:**
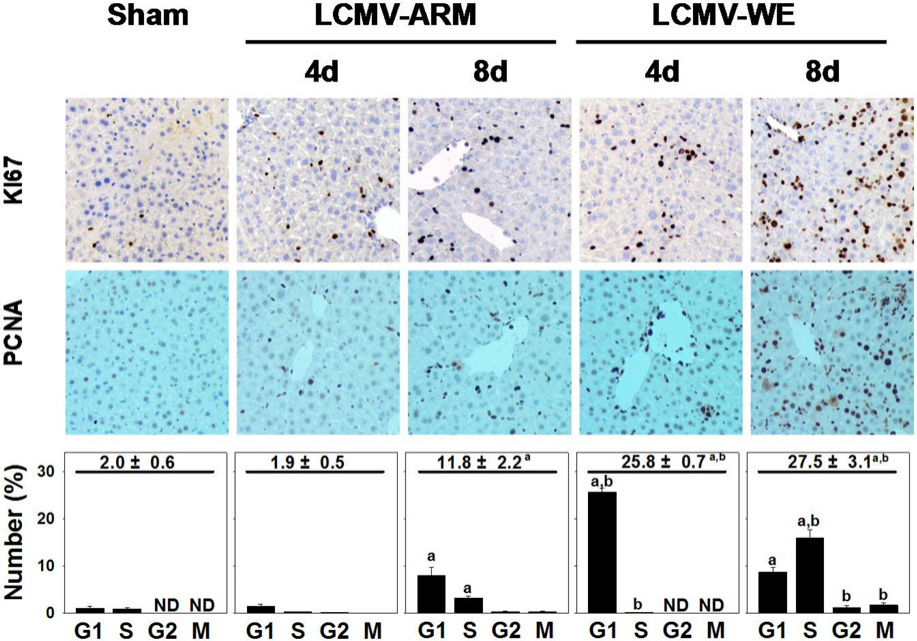
LCMV-WE infection causes cell cycle abortion in hepatocytes. Immunohistochemistry for Ki-67 (200x), proliferating-cell-nuclear-antigen (PCNA, 200x) and quantitative analysis were performed as described in Materials and Methods. Quantitative PCNA summarizes the total % of cells in interphase (upper value), as well as at the individual cell cycle stages (i.e., G_1_, S, G_2_, M). Quantitative data are means ± SEM (n = 4–7).^a^, *P*<0.05 compared with sham infection;^b^, *P*<0.05 compared with LCMV-ARM infection. Data are representative of two experiments performed with liver sections from three mice in each group.

Previous studies in rhesus macaques infected with LCMV-WE [7–9], or in marmosets infected with LASV [46] have indicated that disease progression correlated with hepatocyte proliferation, which is also in line with previous observations in fatally-infected LF patients [11]. To assess proliferation here, liver sections collected on day 4 (no elevation of ALT in serum samples) and day 8 (the highest level of ALT in sera) after infection were stained for Ki-67 and PCNA markers of proliferation (Fig 3).

LCMV infection increased the number of hepatocytes positively stained for Ki-67 and PCNA, indicative of entry into the cell cycle. Notably, LCMV-WE induced more robust proliferative responses in comparison with LCMV-ARM. PCNA staining revealed more cells in interphase, but the effect was much more robust in livers from LCMV-WE infected mice. For example, on day 4 after LCMV-WE infection, ~25% of hepatocytes were positive for PCNA staining (i.e., not G_0_). In contrast, only ~2% of hepatocytes were PCNA positive in LCMV-ARM-infected livers at this time point (Fig 3, lower panels). Eight days after infection, both strains showed increased numbers of proliferating cells in the liver, but the effect was 2-fold stronger in LCMV-WE-infected liver sections. Liver weight to body weight ratios were ~5% in control non-infected mice. LCMV infection with both strains significantly increased liver mass by ~20%. Interestingly, although LCMV-WE infection more robustly increased the number of proliferating cells, it did not significantly increase liver mass after comparison with LCMV-ARM (not shown).

The results from Fig 3 suggest that LCMV-WE infection stimulated a proliferative response in the liver. This induction may also up-regulate expression of more embryonic genes in hepatocytes (e.g., *Dag1*), as well as non-conventional receptors for LCMV and LASV. Therefore, to determine if hepatocyte proliferation itself was sufficient to induce these receptors, the effect of 70% partial hepatectomy (PHx) on expression of these receptors was determined (Fig 2B). As is well-known for this paradigm, PHx rapidly induced a regenerative response (hyperplasia) in the remnant liver, which peaked 48 h after surgery, with 98 ± 1% of hepatocytes positive for PCNA staining [56–58]. While PHx, similar to LCMV infection, slightly up-regulated expression of *Tyro-3* and *LSECtin* in liver, PHx did not affect expression of *Axl-1* mRNA, which was almost 6-fold higher in LCMV-WE-infected livers at day 8 after infection (Fig 2A).

### Pro-inflammatory cytokine and cell cycle genes in liver of LCMV-infected mice

**Fig 4:**
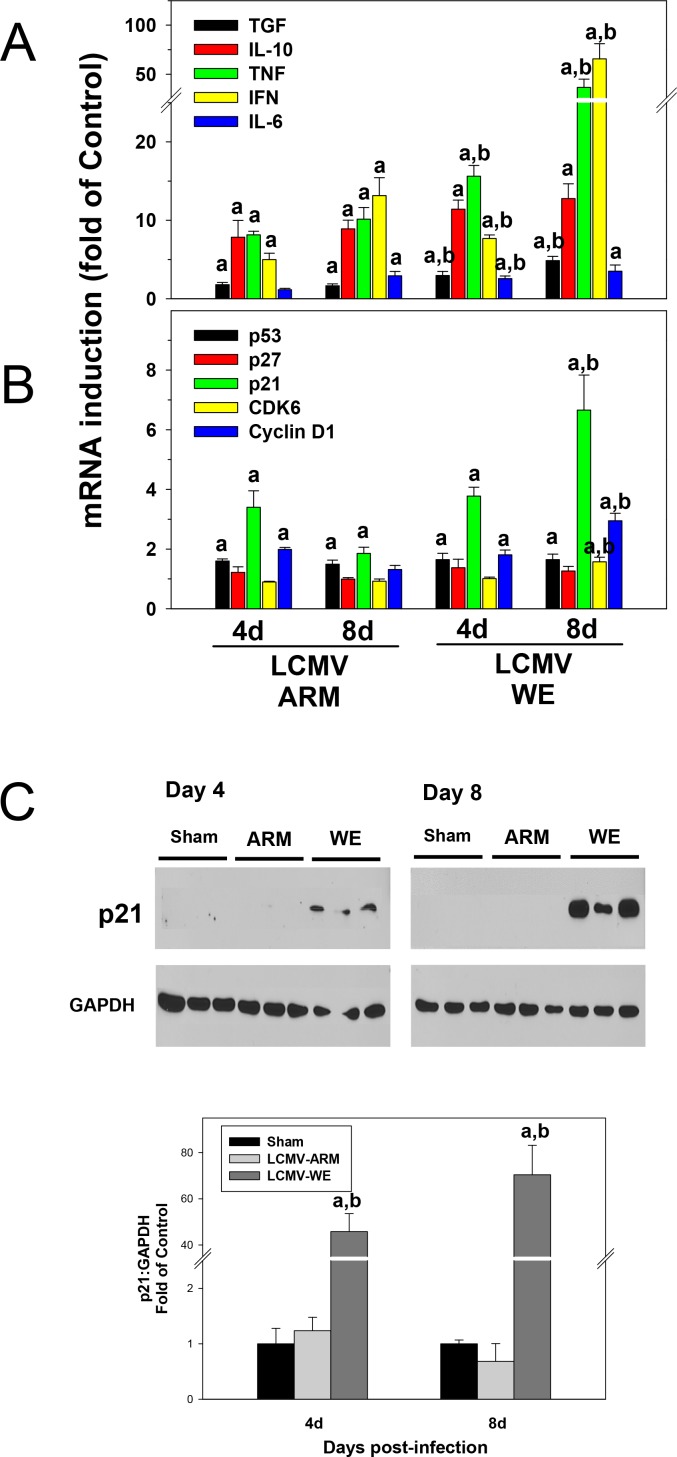
Proinflammatory cytokines and cell cycle gene responses in liver of LCMV-infected mice. Hepatic mRNA was extracted from LCMV-infected mice and real-time RT-PCR for inflammatory (Panel A) and cell cycle (Panel B) genes was performed as described in Materials and Methods. Western blot analysis of p21 protein expression (Panel C) was determined as described in Materials and Methods. GAPDH was used as a loading control. Representative bands (upper) and quantitative densitometry (lower) are shown. Quantitative data are means ± SEM (n = 4–7).^a^, *P*<0.05 compared with sham infection;^b^, *P*<0.05 compared with LCMV-ARM infection. Data are representative of two experiments performed with liver extracts collected from three mice in each group.

Early studies implicated gut-derived microbial components, lipopolysaccharide (LPS), in induction of pro-inflammatory cytokines (e.g., *Tnf-α* and *Il-6*), mostly by resident macrophages, KCs, and triggering hepatocyte proliferation [59,60]. To determine the effect of LCMV infection on inflammatory cytokine production in mice, the expression of key mediators involved in triggering hepatocyte proliferation and in innate immune responses (*Tnf-α*, *Il-6*, *Tgf-β*, *IFN-γ*, *Il-10*) was measured at the level of mRNA expression (Fig 4A). Among the most differently expressed genes were *Tnf-α* and *Ifn-γ*. The expression of these cytokines was ~2-4-fold higher in LCMV-WE infected livers than that in LCMV-ARM infected livers at the tested time points. Expression of *Il-6* mRNA was modestly up-regulated in LCMV-WE infected mice; still it was statistically different (p<0.05) at early time point in comparison with LCMV-ARM infection. Expression of *Tgf-β*, which is involved in termination of liver regeneration, was elevated at 4 and 8 days after infection. The *Tgf-β* mRNA level was higher in LCMV-WE-infected mice than that in livers of LCMV-ARM-infected animals (p<0.05). The expression of *Il-10*, one of the most important anti-inflammatory cytokines in the liver, was strongly up-regulated after LCMV infection but there were no differences between ARM- and WE-infected mice at the tested time points.

As mentioned above, strong proliferative responses of hepatocytes in livers of LCMV-infected mice did not result in a greater liver mass compared to LCMV-ARM infection. Indeed, 4 days after LCMV-WE infection most hepatocytes appear to be accumulating in G_1_ phase (Fig 3, bottom panel). Based on these results and the lack of difference in liver weight between LCMV-ARM and LCMV-WE infected animals, we hypothesized that although LCMV-WE infection induced more cells into interphase, cell cycle is aborted or incomplete. Therefore, the expression of key regulators of entrance and progression through the cell cycle was determined in LCMV-infected livers at the level of mRNA expression by qRT/PCR.

As seen in Fig 4B, we observed differences in mRNA expression of the cell cycle regulators in liver samples from mice infected with LCMV-WE and LCMV-ARM. For example, cyclin D1, an important factor for initiation of DNA synthesis, was not significantly affected by LCMV-ARM at day 8 after infection, but was induced by LCMV-WE. CDK6, a catalytic subunit of the protein kinase complex that is important for cell cycle G_1_ phase progression and G_1_/S transition, was also slightly up-regulated in livers from LCMV-WE mice at this time point. The expression of *p53*, a cycle checkpoint gene, was slightly induced by both LCMV strains. The level of *p27* mRNA encoding a negative regulator of the cell cycle was not significantly changed in liver tissues during LCMV infection. In contrast, the expression of the tumor suppressor *p21* was ~3-fold higher in LCMV-WE infected livers compared to LCMV-ARM infected livers at the 8 day time point. Since *p21* mRNA was among the most differentially affected cell cycle gene in LCMV-WE-infected samples, Western blot analysis was performed to confirm these results. As seen in Fig 4C, *p21* was barely detectable in livers from sham or LCMV-ARM-infected mice 4 and 8 days after infection. However, the induction of p21 by LCMV-WE at the protein level was even more robust than that observed with mRNA and peaked on day 8 (Fig 4C).

### LCMV-WE infection induced apoptosis in liver and expanded population of hepatic progenitor cell population

Hepatocytes have employed a variety of response pathways to protect integrity of their genome via proper coordination of these responses with cell cycle progression and apoptosis. There is a growing body of evidence that virus infection can alter host mitogenic signaling and induce cell cycle arrest [61–64]. In liver, IFN-γ is a key regulator of cell cycle progression that can arrest hepatocyte proliferation in both G_1_ and S, in part via inducing p21. These arrested cells are becoming more sensitive to cell death via induction of apoptosis [65].

**Fig 5:**
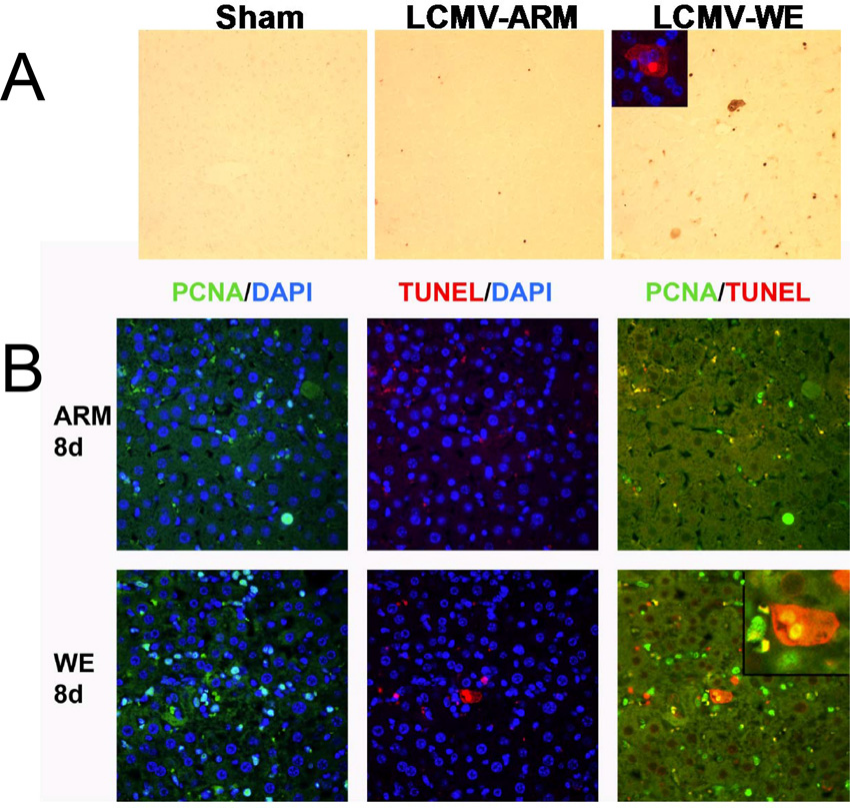
Proliferation and apoptosis overlaps in livers from LCMV-WE-infected mice. Animal groups are as described in Fig 1. Immunohistochemical detection of apoptosis (TUNEL) and proliferation (PCNA) staining were performed as described in Materials and Methods. 4',6-diamidino-2-phenylindole (DAPI, blue) nuclear staining was used as a counterstain for immunofluorescent techniques. Panel A shows representative photomicrographs (200x) depicting apoptosis; the inset (400x) depicts immunofluorescent staining for non-traditional cytosolic TUNEL staining in livers from LCMV-WE infected mice. Panel B shows representative photomicrographs (200x) depicting double immunofluorescent staining for PCNA (left; green) and TUNEL (middle; red) and merged (right; yellow) in livers from LCMV-infected mice. The inset depicts double-immunofluorescent staining in a cell with non-traditional cytosolic TUNEL staining in livers from LCMV-WE infected mice (see also inset for Panel A).

As mentioned above, *Ifn-γ* and *p21* where induced to a greater extent in livers from LCMV-WE infected mice than in those from LCMV-ARM infection (Fig 4). Therefore, the effect of LCMV infection on apoptosis was determined by TUNEL staining. As seen in Fig 5A (upper panels), hepatocytes staining positive for TUNEL were rare in sham controls. While LCMV-ARM infection slightly increased the number of TUNEL-positive cells, this effect was much more pronounced in LCMV-WE-infected liver tissues. Furthermore, whereas the increase in TUNEL signals in LCMV-ARM-infected livers was similar at day 4 and 8 after infection, the number of TUNEL-positive cells in LCMV-WE-infected livers was ~2-fold higher 8 days after infection compared to the day 4 time point (not shown). Interestingly, LCMV-WE infection also caused appearance of non-traditional TUNEL-positive cells, in which the cytosol of oncotic cells was positively stained for TUNEL (Fig 5A, inset). In addition, liver sections were co-stained for TUNEL and PCNA. Hepatocytes with positively stained cytosol for both markers were detected in livers of LCMV-WE-infected mice (Fig 5A and 5B, inset). These double-positive hepatocytes were not found in LCMV-ARM-infected liver sections.

**Fig 6:**
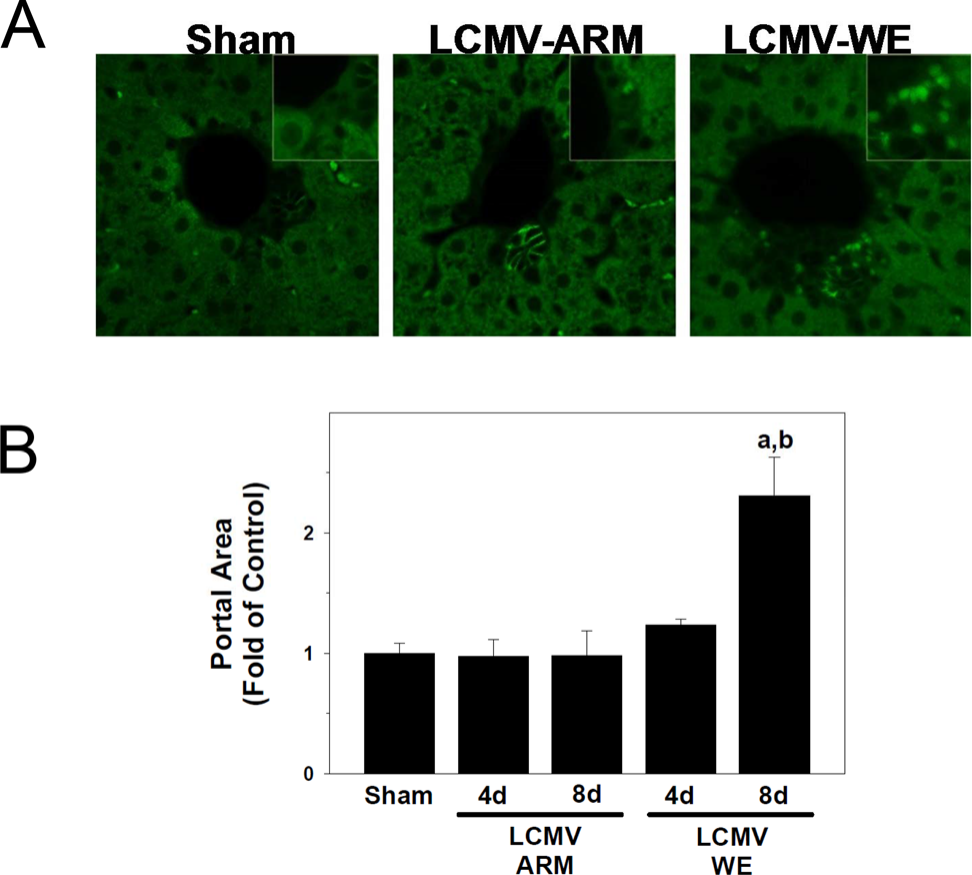
LCMV-WE causes expansion of progenitor cells in portal regions. Animals and treatments are as described in Fig 1 and Materials and Methods. Immunofluorescent detection of A6-positive progenitor cells was performed as described in Materials and Methods. The estimation of the cellular area of the portal tract was determined by image-analysis as described in Materials and Methods and reported as fold of control (sham). Panel A shows representative photomicrographs (400x) of A6 staining in livers from sham or LCMV-infected mice. Insets show a blow-up of the A6-positive cells (or the equivalent area). Panel B shows quantiative determination of portal area size. Quantitative data are means ± SEM (n = 4–7).^a^, *P*<0.05 compared with sham infection;^b^, *P*<0.05 compared with LCMV-ARM infection.

It is well established that when mature hepatocytes fail to properly proliferate (e.g., blocking hepatocyte proliferation by 2-acetylamino-fluorene prior to PHx in rats), a 2^nd^ line of defense is activated in the liver causing an induction of oval cells. These multipotent progenitor cells, typically located within the periportal region, produce alpha-fetoprotein (A6 antigen) and can become mature hepatocytes within a week of activation [66–68]. As it was shown in Fig 3, although more cells entered interphase after LCMV-WE infection, this cell cycle appeared to be aborted or incomplete. To determine if the failure of LCMV-WE-infected hepatocytes to complete cell cycle activated oval cells, we compared expression of A6 antigen in liver sections from LCMV-infected mice (Fig 6). In liver sections from sham-infected animals, the expression of A6 antigen was localized among biliary epithelial cells (Fig 6A). Infection with LCMV-ARM did not alter this pattern of A6 antigen expression. In contrast, the expansion of A6 positive cells was clearly detectable in periportal areas of liver sections from LCMV-WE-infected mice (Fig 6A, inset). This increase in periportal cellularity induced by LCMV-WE infection caused portal expansion and increased the relative size of the portal areas (Fig 6B). This effect was most pronounced 8 days after LCMV-WE infection.

## Discussion

The liver is the major target for conventional hepatotropic viruses and for viruses that cause VHFs, severe systemic diseases with a high fatality rate. For many of these diseases (e.g., Yellow fever, Ebola and Marburg HFs, LF), the liver is not only the major target for the infection, but also involved in pathogenesis and systemic breakdown, leading to a fatal outcome. In spite of some limitations, LCMV infection in mice has been used for decades as a model mimicking CD8+ T cell immunopathology of HCV infection in humans. This model has been helpful to identify several genes that are important for control of LCMV hepatitis in mice and can be potential targets for HCV therapeutic interventions in humans [30].

LCMV infection in rhesus macaques [7,8], captive tamarinds and marmosets [69,70], as well as LASV infection in humans [10,11] and in non-human primates (review by [71]), causes fatal hepatitis with similar clinical and pathophysiological manifestations. Based on these observations, infection of rhesus macaques with viscerotropic (WE) strain of LCMV is considered to be a surrogate model of LF, the most prevalent VHF after Dengue Fever [9,72]. During the first days after infection, LCMV-WE altered expression of genes involved in fatty acid and glucose metabolism, resembling a transcriptome profile of a starvation state with a strong up-regulation of markers for liver regeneration [7,8,34]. These results are in good accordance with hepatocellular regeneration observed in humans fatally infected with LASV [11].

In this study, we used a murine model of LCMV infection to gain insight into mechanism(s) behind the hepatic proliferative responses, and the potential role of hepatocyte hyperplasia in the pathogenesis of LF. In spite of minimal histological findings in LCMV-WE- and LASV-infected liver tissues, a strong proliferative response was one of the major features observed in experimental animals and in LF patients. A rodent model of PHx proposed by Higgins and Anderson in 1931 [73] is one of the best models of liver regeneration. This well-orchestrated and multi-player process seems to be initiated by cytokines (TNF-α, IL-6) released after exposure of KCs to elevated concentrations of gut-derived bacterial LPS [56–58] and induces the expression of more than 40 immediately-early genes that are latent in the quiescent liver. Indeed, here we observed high levels of IL-6 and soluble receptors for IL-6 and TNF-α in plasma of LCMV-WE-infected rhesus macaques [7,8]. Notably, elevated levels of these markers in plasma, as well as Ki-67 positive staining of liver biopsy samples, strongly correlated with clinical manifestations and outcome of the disease. Similar observations were made in non-human primates infected with LASV [71]. In all these models, the proliferative responses were clearly linked with viral burden in the liver. In infected monkeys, Ki-67 staining and other biomarkers of liver involvement were also correlated with clinical manifestations of the disease; while in a mouse model the proliferative response peaked transiently on day 8–10 after infection and quickly declined as soon as virus load in liver was effectively controlled. In this study we demonstrated that replication of LCMV-WE (but not LCMV-ARM) shared the hallmarks of arenavirus-induced hepatitis in non-human primates, namely elevated transaminase levels, strong up-regulation of TNF-α, and induction of liver cell proliferation. We observed only modest up-regulation of IL-6 mRNA expression in LCMV-WE-infected mice.

The second notable observation was that most hepatocytes entering cell cycle remained in interphase and appeared to accumulate at cell cycle transition points (e.g., G_1_/S), suggesting that cell cycle was not completed. In support of this notion, we observed strong induction of *Inf-γ* and *p21* mRNA. Notably, clear differences between LCMV-ARM- and LCMV-WE-infected liver samples were seen in expression of the cell cycle checkpoint protein, p21. This protein restricts interphase progression by causing cell cycle arrest (e.g., at G_1_/S). Although the activity of p21 is controlled post-translationally as well as transcriptionally [74], the robust induction in expression observed here (Fig 4) indicates transcriptional regulation of p21 (most likely via IFN-γ). Thus, this regulation of p21 contributes, at least in part, to failed progression through the cell cycle in response to LCMV-WE infection.

LCMV-WE also induced expression of cyclin D1, especially at day 8 after LCMV-WE infection of mice. Interestingly, in HeLa cells infected with LCMV, cyclin D1 was down-regulated during the first 24 hours after infection but increased dramatically at a later stage of the infection [75]. It has been also shown that LCMV Z protein over-expression down-regulates translation of cyclin D1. This cell cycling factor is required for the G_1_/S transition, and if its production is depressed, infected cells remain in G_1_ arrest. This mechanism also can be involved in slow proliferative responses of T cells and contribute to a viral strategy for establishing chronic infection [75].

In addition, we have shown (Fig 5) TUNEL-positive cells that were also positive for PCNA in animals infected with LCMV-WE, suggesting that hepatocytes with aborted cell cycle were subjected to apoptosis. The finding that cell apoptosis was increased in tandem with proliferation (and potentially as a result of aborted proliferation) may explain why the outcome of infection with a non-cytopathic virus (e.g., LCMV-WE, LASV) can correlate with AST/ALT plasma transaminase values. Certainly, the interplay between acute and chronic infections, proliferation, immune responses, liver pathology and cell cycle regulating factors will be an intriguing area for future research.

Oval cells expand in the liver when hepatocytes fail to respond sufficiently to a proliferative stimulus. As far as LCMV-WE-infected hepatocytes failed to complete the cell cycle, we observed activation of oval cells in portal regions of LCMV-WE-infected liver sections stained for A6 antigen (Fig 6). Activation of oval cells can also contribute to a higher viral burden of LCMV-WE infection in liver. Functional hepatic α-DG expression was greatest at the late gestational and neonatal time points in comparison with mature hepatocytes in adult mice [51], suggesting that progenitor cells (e.g., oval cells) express high levels of functional α-DG, the major cell receptor for LCMV and LASV.

While properly glycosylated α-DG receptor for LCMV and LASV is not expressed in terminally differentiated hepatocytes (due to down-regulation of LARGE and possibly other glycosyltransferases), hepatocytes are still effectively infected with these viruses. Moreover, viral load in the liver perfectly correlates with clinical and biological markers of disease progression. In this study we have found that LCMV infection in C57BL/6J mice induces expression of additional receptors for LCMV and LASV, with AXL-1 being the most responsive protein (Fig 2). Similar to α-DG, AXL-1 transduces signals from the extracellular matrix into the cytoplasm. AXL-1 (TAM family of receptor tyrosine kinases) interacts with its ligand GAS6 (growth-arrest-specific protein 6) before it can be activated. AXL-1 activation correlates with proliferation and plays a critical role in the progression of cancer [76]. Interestingly, activation of AXL-1 protects hepatic oval cells from apoptosis and the induction of *Axl-1* expression may be linked to the observed induction of proliferation in hepatocytes and expansion of oval cells.

Recently, Fletcher et al. [77] showed that pretreatment of polarized hepatocytes with TNF-α (or with culture medium from LPS-stimulated macrophages) resulted in disruption of tight junction integrity and promoted entry and replication of rVSV pseudo-particles bearing the surface GP of LASV. Taking into consideration strong induction of TNF-α expression in livers of LCMV-WE-infected mice (Fig 4A), we cannot exclude the possibility that this mechanism potentially contributes to a higher viral load in LCMV-WE-infected livers. However, the concentration of TNF-α in those *in vitro* experiments was supra-physiologic. In addition, in preliminary experiments we failed to reproduce these results using infection of polarized and TNF-treated HepG2 cells with LCMV (data not shown).

In conclusion, using a murine model of LCMV infection, we demonstrated a new pathway for arenavirus-induced liver pathology. In this model, infection of mice with the viscerotropic WE strain, but not the neurotropic ARM strain, induced transient liver pathology resembling fatal hepatitis caused by LCMV in non-human primates and LASV hepatitis in humans. In these models, the viral load in the liver was correlated with elevated levels of aminotransferases (AST/ALT) in plasma, with strong activation of pro-inflammatory cytokines (TNF-α, IL-6, IFN-γ), and with activation of hepatocytes proliferation. We have shown here, that upon activation, cell cycle was aborted and more than 27% of hepatocytes were arrested in G_1_/S transition in LCMV-WE-infected livers. Aborted hepatocyte proliferation correlated with elevated levels of cyclin D1 and CDK6 and aborted proliferation also resulted in apoptosis and stimulation of oval cells. Furthermore, LCMV infection also stimulated expression of non-conventional receptors for LCMV and LASV. The role of these receptors, oval cells, and TNF-α-inducible disruption of tight junction integrity should be further elucidated to answer the question why mature hepatocytes remain highly permissive for pathogenic arenaviruses, LCMV-WE and LASV. We speculate that a cell cycle arrest hepatocyte proliferation model of arenavirus-induced hepatitis can be applicable for other non-conventional viral hepatitis (e.g., YF, Ebola, Marburg).
